# Photolysis of Dissolved Organic Matter over Hematite Nanoplatelets

**DOI:** 10.1021/acs.est.3c08752

**Published:** 2024-01-31

**Authors:** Xiaopeng Huang, Duo Song, Qian Zhao, Robert P. Young, Ying Chen, Eric D. Walter, Nabajit Lahiri, Sandra D. Taylor, Zheming Wang, Kirsten S. Hofmockel, Fernando Rosario-Ortiz, Gregory V. Lowry, Kevin M. Rosso

**Affiliations:** † Physical and Computational Sciences Directorate, Pacific Northwest National Laboratory, Richland, Washington 99352, United States; ‡ Earth and Biological Sciences Directorate, Pacific Northwest National Laboratory, Richland, Washington 99352, United States; § Environmental Molecular Sciences Laboratory, Pacific Northwest National Laboratory, Richland, Washington 99352, United States; ∥ Civil and Environmental Engineering, Carnegie Mellon University, Pittsburgh, Pennsylvania 15213, United States; ⊥ Center for Environmental Implications of Nano Technology (CEINT), Carnegie Mellon University, Pittsburgh, Pennsylvania 15213, United States; # Department of Civil, Environmental, and Architectural Engineering, University of Colorado, Boulder, Boulder, Colorado 80309-0607, United States; ∇ Environmental Engineering Program, University of Colorado, Boulder, Boulder, Colorado 80309-0428, United States

**Keywords:** dissolved organic matter, hematite, reactive oxygen species, photodegradation, interfacial electron transfer

## Abstract

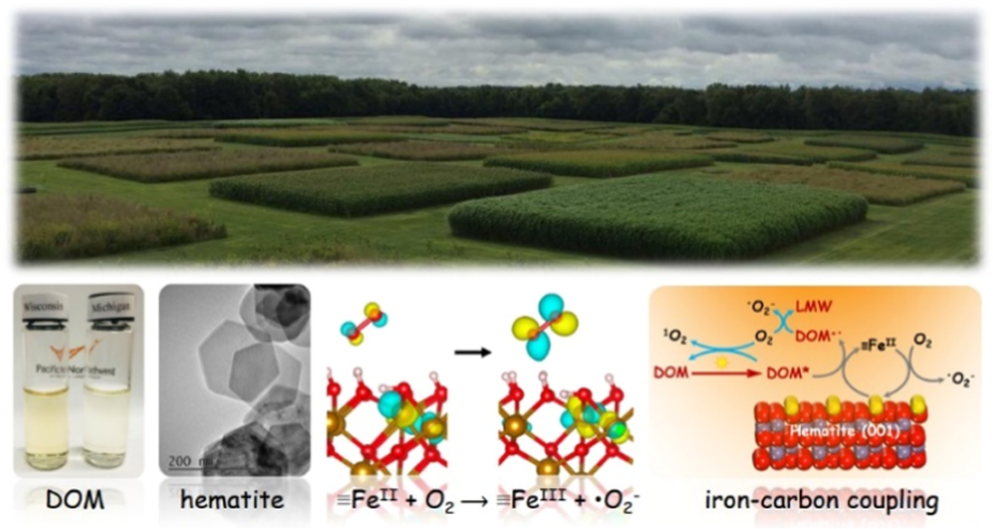

Solar photoexcitation of chromophoric groups in dissolved organic matter (DOM), when coupled to photoreduction of ubiquitous Fe(III)-oxide nanoparticles, can significantly accelerate DOM degradation in near-surface terrestrial systems, but the mechanisms of these reactions remain elusive. We examined the photolysis of chromophoric soil DOM coated onto hematite nanoplatelets featuring (001) exposed facets using a combination of molecular spectroscopies and density functional theory (DFT) computations. Reactive oxygen species (ROS) probed by electron paramagnetic resonance (EPR) spectroscopy revealed that both singlet oxygen and superoxide are the predominant ROS responsible for DOM degradation. DFT calculations confirmed that Fe(II) on the hematite (001) surface, created by interfacial electron transfer from photoexcited chromophores in DOM, can reduce dioxygen molecules to superoxide radicals (^•^O_2_^–^) through a one-electron transfer process. ^1^H nuclear magnetic resonance (NMR) and electrospray ionization Fourier-transform ion cyclotron resonance mass spectrometry (ESI-FTICR-MS) spectroscopies show that the association of DOM with hematite enhances the cleavage of aromatic groups during photodegradation. The findings point to a pivotal role for organic matter at the interface that guides specific ROS generation and the subsequent photodegradation process, as well as the prospect of using ROS signatures as a forensic tool to help interpret more complicated field-relevant systems.

## Abstract 2

The photocatalytic ferrous iron pool that was created at hematite surfaces coated by organic matter can reduce oxygen to reactive oxygen species for photodegradation.

## Introduction

The mineral–organic interface plays an important but incompletely understood role in the transformation dynamics, storage, and turnover of natural organic matter in the environment, including soil organic matter (SOM) and dissolved organic matter/carbon (DOM/DOC).^1^ For instance, binding to minerals is widely assumed to protect SOM from degradation, promoting the preservation of carbon in soil and sediments.^2,3^ However, in the presence of light, e.g., at the soil surface or in the euphotic zone of aquatic environments, strong binding to ubiquitous photoredox-active nanoparticles such as Fe(III)-oxides can catalyze SOM and DOM degradation.

DOM–mineral interactions are important in both carbon and metal biogeochemical cycling in aquatic environments. DOM is a polydisperse mixture of macromolecules derived from biological sources^4^ and composed of a varied collection of organic molecules that often include chromophoric groups. DOM is a critical component in aquatic systems because it can bind to iron oxide surfaces, where it can act as a photosensitizer, promoting interfacial electron transfer that reduces the mineral Fe(III) to Fe(II). This has two important consequences. First is that this becomes an important source of aqueous soluble bioavailable Fe(II), with important impacts on iron biogeochemical cycling.^5^ Second, this valence cycling of iron stimulates production of reactive oxygen species (ROS), opening a pathway to organic matter degradation.^6^ Adsorbed terrestrially derived DOM that enables photoinduced iron redox cycling has been shown to exhibit enhanced bleachability.^7^ This ROS pathway can consistently degrade DOM in surface waters, thus impacting ocean organic compounds and biota^8^ and ultimately playing a major role in the dynamics of the total DOM pool and the global carbon cycle.^9^ In arctic lakes and rivers, Cory et al. found that the rate of photochemical oxidation of dissolved organic carbon (DOC) exceeds rates of DOC respiration and accounts for 70 to 95% of total DOC processed in the water column.^9^ They demonstrated that the photochemical processing of DOC is an important component of the arctic carbon budget, accounting for about one-third of the total CO_2_ released from surface waters at the basin scale.

While photochemical processing of DOM in the water column of arctic lakes and river bodies has been studied at the basin scale,^9^ the photodegradation pathway of terrestrially derived SOM (i.e., the source of the DOM) is less well understood. In terrestrial ecosystems with fluctuating O_2_ availability, previous studies found that SOM can be degraded as electron donors to stimulate microbial Fe reduction under an anaerobic condition.^10,11^ Reduced Fe(II) oxides can be further oxidized to Fe(III) under subsequent aerobic conditions^12,13^ Fe(II) oxidation promotes SOM degradation via ROS oxidation and acidification.^13^ Elucidating the processes affecting SOM is important because approximately 0.25 Pg of DOC is transported annually from soils to the oceans through aquatic systems,^14,15^ The poor understanding of photodegradation of SOM is partly due to the compositional complexity of SOM, which is difficult to characterize systematically at the molecular level, even with state-of-the-art analytical capabilities. But, it is also due to its spatial and seasonal heterogeneity, such as variable soil pore volumes, precipitation regimes, and hydrologic cycling. Additionally, the critical role of iron oxides in soil, especially highly mobile and chemically reactive iron oxide nanoparticles, on the photochemical processing of SOM/DOM at the terrestrial source is not understood. Understanding this process will improve estimates of SOM/DOM degradation rates in the photic zone of terrestrial environments.

The present study focuses on the photodegradation pathways of DOM derived from SOM in terrestrial ecosystems, specifically from agricultural soils, which is one of the origins of DOM in aquatic ecosystems. We recently examined DOM photodegradation on hematite nanoplatelets functionalized by oxalate (a model photosensitive DOM compound).^7^ In this case, illumination caused adsorbed oxalate to undergo ligand-to-iron charge transfer, yielding Fe(II) and the carboxyl anion radical, which subsequently reduced molecular oxygen into hydrogen peroxide, thus initiating hydroxyl radical production via heterogeneous Fenton reactions that degrade DOM. However, it remains unclear if this same ROS reaction pathway operates in the absence of adsorbed oxalate. If chromophoric groups in natural DOM are capable of initiating similar ROS production reactions, other radicals may become important in the absence of the carboxyl anion radical derived from oxalate. The radicals that form from DOM in the absence of the oxalate would be more representative of those found in natural systems.

Hematite {001} facets possess a relatively low surface energy and are one of the most commonly exposed surfaces of natural hematite.^16^ We examined the photodegradation of DOM samples from two agricultural soils with contrasting DOM composition in the presence of hematite nanoplatelets with {001} exposed facets without sensitization by oxalate. Detailed characterization of ROS generation and DOM carbon chemistry before and after photodegradation was performed by using a combination of electron paramagnetic resonance (EPR), X-ray photoelectron spectroscopy (XPS), nuclear magnetic resonance (NMR), and electrospray ionization Fourier-transform ion cyclotron resonance mass spectrometry (ESI-FTICR-MS). The associated interfacial electron transfer processes were assessed by using first-principles electronic structure calculations based on plane-wave density functional theory (DFT) to study the one-electron transfer process that reduces dissolved oxygen molecules by surface Fe(II) polarons to form superoxide radicals. The findings broaden our mechanistic understanding of potential photodegradation pathways of DOM from agricultural soils that control its lifetime in terrestrial and aquatic ecosystems and could ultimately help improve predictive modeling of terrestrial-atmosphere carbon flux in global carbon biogeochemical cycling.

## Experimental Methods

### Synthesis of Hematite Nanoplatelets

The synthesis method used for the hematite nanoplatelets (HNPs) is described in previous work,^17^ and the same material has been used in prior reactivity studies.^7^ Briefly, iron chloride (4.0 mmol) was dissolved in ethanol (40 mL) and water (2.8 mL). Then, sodium acetate (3.2 g) was added. The mixture was reacted at 180 °C for 12 h. The synthesis protocols are provided in detail in Text S1. The morphology of the resulting HNPs entails 87% of the geometric surface area comprised of (001) basal surfaces and 13% as (012) as edge surfaces.^7^

### Natural DOM Extraction

DOM was extracted from surface soils sampled from Wisconsin soil (DOM_WS_) and Michigan soil (DOM_MS_) at the Great Lakes Bioenergy Research Center. Briefly, soil was collected from the top 15 cm below the surface, passed through a 2 mm sieve, and air-dried at room temperature. Dry soil was weighed in polypropylene centrifuge tubes and mixed with water at a 1:10 w/v ratio. The supernatant was collected and filtered with 0.2 μm syringe filters, finally yielding dissolved organic matter extracted from Wisconsin soil (DOM_WS_) and Michigan soil (DOM_MS_), respectively. The detailed extraction methods are given in Text S2.

### Photodegradation Experiments

Photodegradation experiments were set up according to our previous study that were described in detail in Text S3.^7^ Briefly, 10 mg of HNPs was dispersed in a 10 mL of DOM aqueous solution in a 200 mL cylindrical Pyrex vessel. The mixture was stirred with a Teflon-coated magnetic stir bar with a magnetic stirrer mixer. The suspension was illuminated by a 200 W xenon arc lamp, simulating the solar spectrum that induces photodegradation reactions.

### EPR Spectroscopy

EPR was used to detect and quantify the radical species. All EPR measurements were performed on a Bruker ELEXSYS E580 spectrometer equipped with an SHQE resonator with an optical access port for *in situ* illumination. The same illumination source was induced into the sample cavity via an optical fiber to illuminate the capillary. A capillary with an ID of 0.8 mm and an OD of 1 mm was used to hold the solution in the EPR cavity with both ends sealed by Critoseal. Kinetic measurements were performed by recording EPR spectra continually before, during, and after illumination with a sweep time of 20.97 and 16 scans at microwave power of 20 mW. The procedures were described in detail in Text S4.

### Computational Details

Computational chemistry was used to test the veracity of the one-electron transfer processes inferred from experiments. Density functional theory (DFT)^18^ calculations were performed with the pseudopotential plane-wave NWPW module^19^ in the NWChem software package.^20,21^ The Perdew–Burke–Ernzerhof (PBE) functional^22^ was used to account for the exchange correlation energy in geometry optimizations. The on-site Coulombic (*U*) and exchange (*J*) constants were introduced in the energy functional^23^ to correct the electron self-interaction error. In this work, *U* and *J* were chosen as 4 and 1 eV respectively, which were found by comparing the DFT + *U* calculated properties using different values (band gap, local spin moment, unit cell volume, etc.) to experimental values.^24^ These parameters were applied only to the d-orbitals of the iron elements. The hybrid functional HSE^25−27^ was applied to calculate the projected density of states for all structures. In our plane-wave calculations, valence electron interactions with the atomic cores were approximated with generalized norm-conserving Hamann pseudopotentials^28^ for O and H, whereas for Fe, we used norm-conserving Troullier–Martins pseudopotentials,^29^ which contain 4s, 4p, and 3d projectors and a semicore correction. All the pseudopotentials were modified to the separable form suggested by Kleinman and Bylander.^30^ Unrestricted calculations were performed since this is a spin-ordered system. The electronic wave functions were expanded using a plane-wave basis with periodic boundary conditions at the Γ-point with a wave function cutoff energy of 100 Ry and a density cutoff energy of 200 Ry.

### XPS Methods

XPS was used to differentiate carbon species before and after the photoreaction on hematite surfaces. X-ray photoelectron spectra were acquired on a Kratos AXIS Ultra DLD instrument equipped with a multichannel detector and a hemispherical analyzer. DOM-coated HNPs were suspended in deionized water and drop casted onto Si wafers containing a 300 nm layer of thermally grown SiO_2_. For the solution-based samples, the supernatant solution was drop-cast onto Au-coated Si wafers. The detailed methods were provided in Text S5.

### Nuclear Magnetic Resonance Spectroscopy

NMR was used for additional DOM compositional characterization before and after photolysis, complementing XPS by focusing on H-bearing speciation. 1D ^1^H NMR measurements were used to characterize the extracted DOM under the following conditions: with or without HNP and with or without exposure to light. Centrifugation and filtration were used to pellet/separate the HNPs from the samples. Measurements were conducted at a regulated temperature of 25 °C using a Bruker Avance III spectrometer operating at a field strength of 17.6 T and equipped with a 5 mm Bruker TCI/CP HCN cryoprobe with Z-gradient. Detailed NMR methods are provided in Text S6.

### FTICR-MS Data Acquisition and Data Analysis

For ultrahigh-resolution chemical characterization of DOM stoichiometry, samples were analyzed using a 12 T FTICR-MS (Bruker-SolariX) with an ESI source. DOM samples were diluted in MeOH at a 1:2 ratio to improve ESI efficiency and then introduced directly to the ESI source with a fused silica tube (30 μm i.d.) at a flow rate of 3.0 μL/min by an Agilent 1200 series pump. The ion accumulation time (IAT) was adjusted between samples to account for different carbon concentrations. The detailed methods were provided in Text S7.

## Results and Discussion

The two DOM samples (DOM_WS_ and DOM_MS_) were characterized before, during, and after light illumination and with and without HNPs present to quantify both the photogeneration of ROS species and the corresponding compositional evolution of the organics. EPR results are presented first, which indicate the ROS species that are forming, and then the inferred photolysis mechanism supported by DFT computations is discussed. The evolution of the organics is then presented based on the integrated XPS, NMR, and ESI-FTICR-MS analytical results.

### Singlet Oxygen and Superoxide Dominate the Photolysis Species

**Figure 1:**
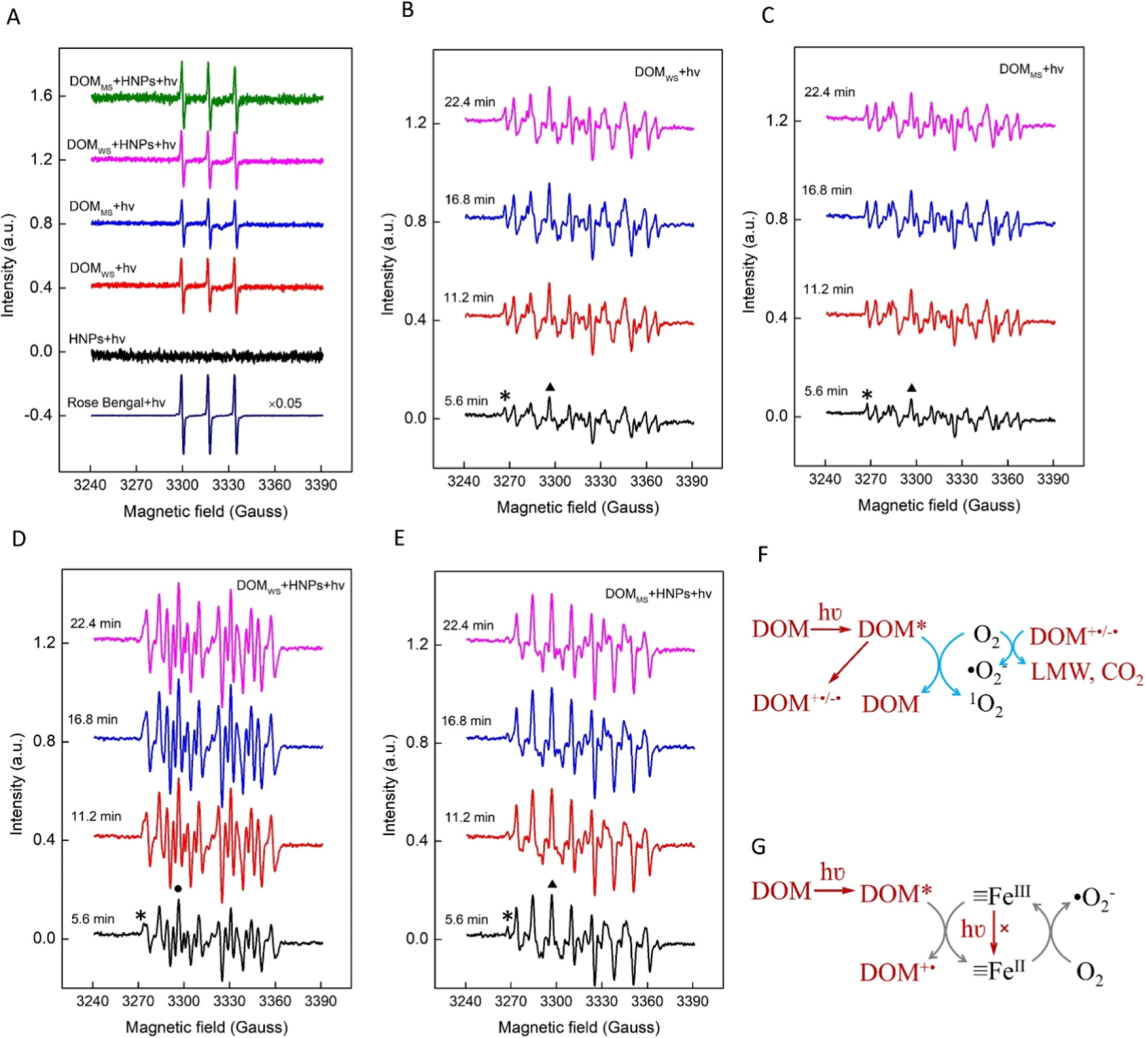
EPR spectra of photolysis of DOM in the absence and presence of hematite nanoplates. (A) TEMP-trapped EPR spectra in the absence or presence of hematite nanoplates. (B) DEPMPO-trapped EPR spectra of photolysis of DOM_WS_ as a function of illumination time. (C) DEPMPO-trapped EPR spectra of photolysis of DOM_MS_ as a function of illumination time. (D) DEPMPO-trapped EPR spectra of photolysis of DOM_WS_ in the presence of hematite as a function of illumination time. (E) DEPMPO-trapped EPR spectra of photolysis of DOM_MS_ in the presence of hematite as a function of illumination time. The symbols star (*), triangle (▲), and dot (●) represent DEPMPO-R, overlapped DEPMPO-OOR and DEPMPO-OOH, and DEPMPO-OR, respectively. The dosage of rose bengal, SOD, and hematite are 40.0 μM, 0.1 g/L, and 1.0 g/L, respectively. The concentration of spin trap TEMP and DEPMPO are 10 mM and 20 mM, respectively. The amount of DOM_WS_ and DOM_MS_ are both 10 μL of the pristine solutions and the pH are 6.38 and 6.28, respectively. Total reaction volume for EPR is 50 μL. (F) Scheme of ROS generation in photolysis of DOM. (G) Scheme of the interfacial iron redox cycle coupled to ROS in photolysis of DOM. “≡” symbol represents the hematite surface.

To isolate the dominant photolytic ROS species with and without HNPs present, various spin trap additives were used in EPR spectroscopy. Experiments were performed by using control measurements based on known ROS-generating compounds. TEMP-trapped EPR spectra were used to detect singlet oxygen (^1^O_2_) (Figure 1A), which reflects the activation of dissolved molecular oxygen to its lowest excited electronic state.^31^ For example, it is known that ^1^O_2_ can be produced during the illumination of the sensitizer rose bengal.^32^ The 2,2,6,6-tetramethyl-1-piperidinyloxyl (TEMP)-trapped EPR detected ^1^O_2_ during illumination of 40.0 μM rose bengal, as indicated by the three-line spectrum with the intensity ratio of 1:1:1, indicating the formation of the spin adduct TEMP–^1^O_2_ (Figure 1A). During the illumination of HNPs alone, no signal of ^1^O_2_ was observed, consistent with its inability to activate molecular oxygen. Illumination of DOM_WS_ and DOM_MS_ in the absence of HNPs produced an EPR spectrum similar to those of rose bengal, indicating that the illumination of the two DOM samples generated ^1^O_2_. The respective amounts of photogenerated ^1^O_2_ furthermore did not detectably change with the addition of the HNPs, indicating that mineral–organic interactions did not inhibit photodegradation and activation of molecular oxygen. Photogenerated ^1^O_2_ is thus an excitation product of our DOM samples alone, consistent with prior work.^33,34^ This includes Zepp et al. in 1977 who first found that solar irradiation of chromophoric DOM generates singlet oxygen ^1^O_2_.^35^

Likewise, we tested several other ROS species using their distinct spectroscopic features (Figure S1). 5-(Diethoxyphosphoryl)-5-methyl-1-pyrroline-N-oxide (DEPMPO) trap is capable of detecting a variety of oxygen-centered and/or carbon-centered free radicals. For example, in control measurements, for DEPMPO-OOH, eight identical lines were observed with an alternate line width, showing the usual shape across the whole EPR spectrum (Figure S1) owing to an exchange between different forms of the DEPMPO-superoxide spin adducts with different hyperfine coupling constants.^36^ Carbon-centered radicals showed 12 peaks with similar intensity (Figure S1). Hydroxyl radical (^•^OH) spin adducts displayed two sets of quartet peaks with an intensity of 1:2:2:1 (Figure S1). We note here that EPR cannot distinguish the hydroperoxyl radical (•OOH) versus superoxide radicals (^•^O_2_^–^), a point we address further below.

When applying the DEPMPO spin trap to our DOM and DOM-NHP systems, we observed a substantial enhancement in ROS generation for our two DOM samples with HNPs present. Measured DEPMPO-trapped EPR spectra are shown in Figures 1B–E and S2. The intensity of the spectra for both DOM_WS_ (Figures 1B,D and S2A) and DOM_MS_ (Figures 1C,E and S2B) with HNPs was much larger than in their absence, revealing that more free radicals were generated with the addition of HNPs (Text S8).

The fitting of component spectra and quenchers were used to determine which radical species were contributing to the observed DEPMPO-EPR signals. Because the spin adducts of DEPMPO-OOH and DEPMPO-OOR are normally coupled together, we added superoxide dismutase (SOD), which can decompose ^•^OOH (*k* = 2.3 × 10^9^ M^–1^ s^–1^),^37^ to differentiate DEPMPO-OOH from DEPMPO-OOR. In all cases, SOD addition significantly reduced EPR signal intensity, indicating that hydroperoxyl radical ^•^OOH (or ^•^O_2_^–^) is an important contributor to the measured DEPMPO-EPR signals.

Although the photodegradation products of DOM were similar for both DOM samples, the presence of HNPs shifted the relative abundance. Table S1 summarizes the resulting EPR simulation parameters for the different radical spin adducts. Photolysis of DOM in the absence of HNPs generated similar proportions of DEPMPO-OOH, DEPMPO-OOR, and DEPMPO-R for DOM_WS_ (40, 30, and 30%, respectively; Figure S3A) and for DOM_MS_ (30, 30, and 40%; Figure S3B). However, in the presence of HNPs, these proportions change substantially; for DOM_WS_, they become 93, 5, and 2% (Figure S3A), and for DOM_MS_, they become 80, 10, and 10% (Figure S3B). What is more, the intensity changes of corresponding ROS in DEPMPO-trapped EPR spectra in the absence and presence of SOD during photolysis of DOM with/without hematite nanoplatelets as a function of illumination time also showed that the DEPMPO-OOH is the major species (Figure S4). Preliminary analyses showed that the presence of HNPs seemed to result in a predominance of hydroperoxyl radical formation. However, the actual species was superoxide radicals, in which the generation mechanism was determined as follows.

Because the hydroperoxyl radical is related to alkyl radicals through the reaction ^•^OOH + R → ^•^OR, it was important to further scrutinize this result by examining its dependence with respect to illumination time, which in all cases showed that ^•^OOH (or ^•^O_2_^–^) dominated and that ^•^OR radicals were relatively minor (Text S8). Therefore, the presence of HNPs substantially shifts the proportions of photolytic radical products for both DOM samples to diminish alkyl radicals and promote formation of the hydroperoxyl radical.

Given the importance of the hydroperoxyl radical, it was important to distinguish ^•^OOH versus ^•^O_2_^–^, which cannot be done with EPR. However, system pH provides an indication of which of these two species should dominate. The relevant equilibrium is ^•^O_2_^–^ + H^+^ ↔ ^•^OOH, p*K*_a_ = 4.9. As the pH values of DOM_WS_ and DOM_MS_ systems are 6.38 and 6.28, respectively, in addition to singlet oxygen ^1^O_2_ as a photolysis product common to both DOM samples, the primary photolytic radical without, but much more so with, HNPs was ^•^O_2_^–^.

It is noteworthy that other common ROS species were not detected. For example, in our DEPMPO-EPR measurements ^•^OH was not detected, despite DEPMPO being a very sensitive ^•^OH spin trap. ^•^OH is common in natural waters where DOM is abundant and in a variety of other natural environments.^38,39^ The ^•^OH was predominant in our prior study of this DOM/HNP system when the HNPs were functionalized by oxalate.^7^ This suggests that DOM photolysis, irrespective of the presence of HNPs, does not directly or indirectly yield ^•^OH with a lifetime that is sufficient to be detected. An example of an indirect pathway would be if DOM photolysis generated hydrogen peroxide (H_2_O_2_), which can lead to ^•^OH production by O–O bond heterolysis of H_2_O_2_, or by Fenton reactions in the presence of dissolved Fe (e.g., Fe(II)). H_2_O_2_ production can occur by a two-electron and two-proton concerted reduction of O_2_, a process unlikely to occur at circumneutral pH but promoted in the presence of oxalate due to photolytic carboxyl anion radical production.^7^ Although photodegradation of DOM has previously been shown to lead to low molecular weight organics such as oxalate^7^ in the current system, this process appears insufficient to produce H_2_O_2_ at concentrations significant enough to yield detectable ^•^OH. Given the oxic conditions of our experiments, the dissolved iron concentrations in our system are expected to be subpicomolar due to the extremely low solubility of hematite at circumneutral pH.^40^ Although we cannot exclude that the steady-state concentration of ^•^OH is kept very low (e.g., 10^–18^–10^–16^ M) by mass transfer limitations,^34^ the above analyses suggest that ^•^OH generation is likely negligible in the current system.

### ROS Generation Mechanisms

DOM with chromophoric functional groups is a light absorber that can be photoexcited to a new electronic state (DOM*). Before it decays back to the ground state, excited triplet DOM* can activate the triplet ground state of molecular oxygen O_2_ to singlet oxygen by energy transfer (Figure 1F). Meanwhile, the photolysis of DOM can also lead to charge-separated intermediate DOM species (DOM^+•/–•^) via intramolecular donor–acceptor electron transfer (e.g., between aromatic ketones and lignin phenols).^41^ Dissolved oxygen can then also react with intermediate DOM^–•^ groups to form superoxide anion radicals as
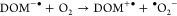


The production of ^•^O_2_^–^ can oxidize some components of the DOM to low molecular weight organics (LMW) and carbon dioxide (CO_2_) (Figure 1F).^41,42^ Other oxygen- or carbon-centered radicals, such as ^•^OOR and ^•^R, are minor species in soil DOM.

**Figure 2:**
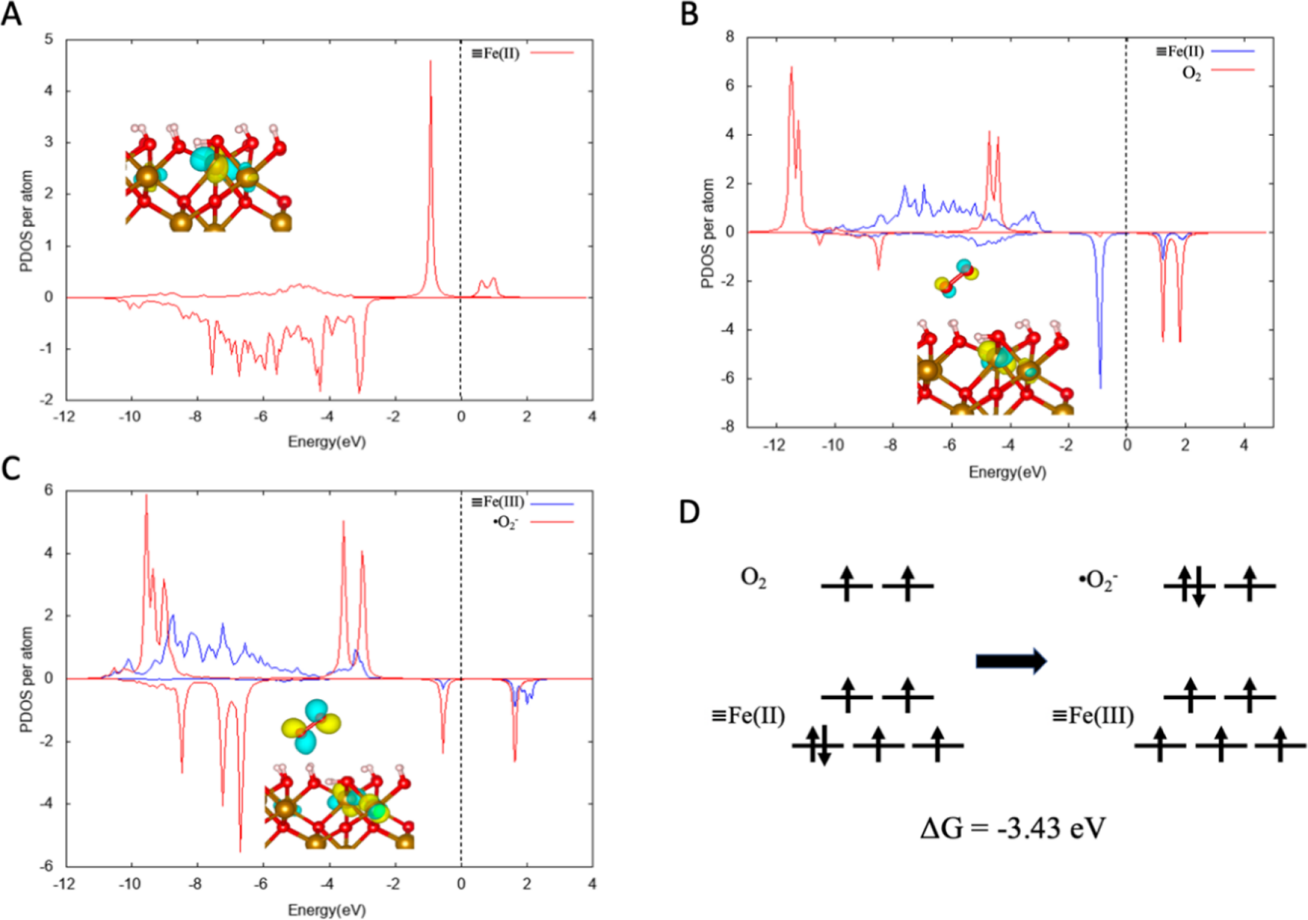
Projected densities of states of (A) surface Fe(II) (B) surface Fe(II) and absorbed O_2_ (C) surface Fe(III) and absorbed ^•^O_2_^–^ (D) The molecular orbital diagrams and the reaction Gibbs free energy for ≡Fe(II) + O_2_ → ≡Fe(III) + ^•^O_2_^–^. In our notation, ≡Fe represents an iron atom on the hematite surface. The inserts in (A–C) are the corresponding surface and molecular structures. The red atoms are O, gold atoms are Fe, and white atoms are H.

The observed enhancement of ^•^O_2_^–^ production in the presence of HNPs suggests an important interaction between photoexcited DOM* and the conduction band of hematite by interfacial electron transfer. Photoexcitation of organic dye molecules adsorbed on hematite surfaces has been shown to be capable of donating electrons to surface iron sites (≡Fe(III)), yielding reactive surface Fe(II) sites.^43,44^ Based on our observations, we hypothesized that these ≡Fe(II) sites are sufficiently reducing to reduce O_2_ at the interface via one-electron transfer, opening another reaction pathway to forming ^•^O_2_^–^ (Figure 1G). To test this hypothesis, we performed density functional theory (DFT) calculations to evaluate the energetics of interfacial electron transfer from ≡Fe(II) on hematite (001) surfaces to adsorbed O_2_. The (001) surface, which is one of the lowest-energy facets of hematite, has been extensively studied in various surface chemistry contexts.^16^ The computed structure and electronic density of states (DOS) for an Fe(II)-like surface site on the (001) surface are shown in Figure 2. Minority spin-up 3d states are observed in the DOS, while the majority states are spin-down 3d states at the upper edge of the valence band (Figure 2A). Figure 2B shows the DOS of surface Fe(II) with absorbed O_2_. After O_2_ adsorption, the spin states of the hematite (001) surface changed to match the different oxygen states as the extra electron on the Fe(II) site couples to the O_2_ π* orbital, ultimately to form Fe(III) and an ^•^O_2_^–^ radical as products (corresponding DOS shown in Figure 2C). Fe(III) spin states facilitate the formation of O 2p states with two occupied majority, one occupied minority, and one empty minority states, indicating that the activated dioxygen was in an −1 reduced valence state of low-spin π*, consistent with the electron configuration of ^•^O_2_^–^. The majority states of the Fe(III) are spin-down, and the spin-up state density is minimal, which agrees with the fact that the Fe(III) is high-spin and Fe cations within planes perpendicular to [001] are coupled spin parallel. The molecular orbital diagram and corresponding DFT-calculated reaction energy (approximately the Δ*G*) for ≡Fe(II) + O_2_ → ≡Fe(III) + ^•^O_2_^–^ is shown in Figure 2D. The calculated Δ*G* was −3.43 eV, indicating that ≡Fe(II) sites on hematite (001) surfaces are thermodynamically capable of reducing dioxygen to superoxide radicals by interfacial electron transfer. Thus, although we expect that Fe(II) concentrations were very low due to the low solubility of hematite, DFT supports the hypothesis that it is an important transient catalytic species at the mineral–organic interface in our system.

### DOM Chemistry Altered by Photolysis

Because DOM exhibits a complex array of functional groups and macromolecular structures, such as carbohydrates, amino sugars, lignin, tannin, and proteins, NMR, FTICR-MS, and XPS were performed to provide complementary information on carbon speciation to understand how the photolysis reactions of our two DOM samples alter DOM composition.

**Figure 3:**
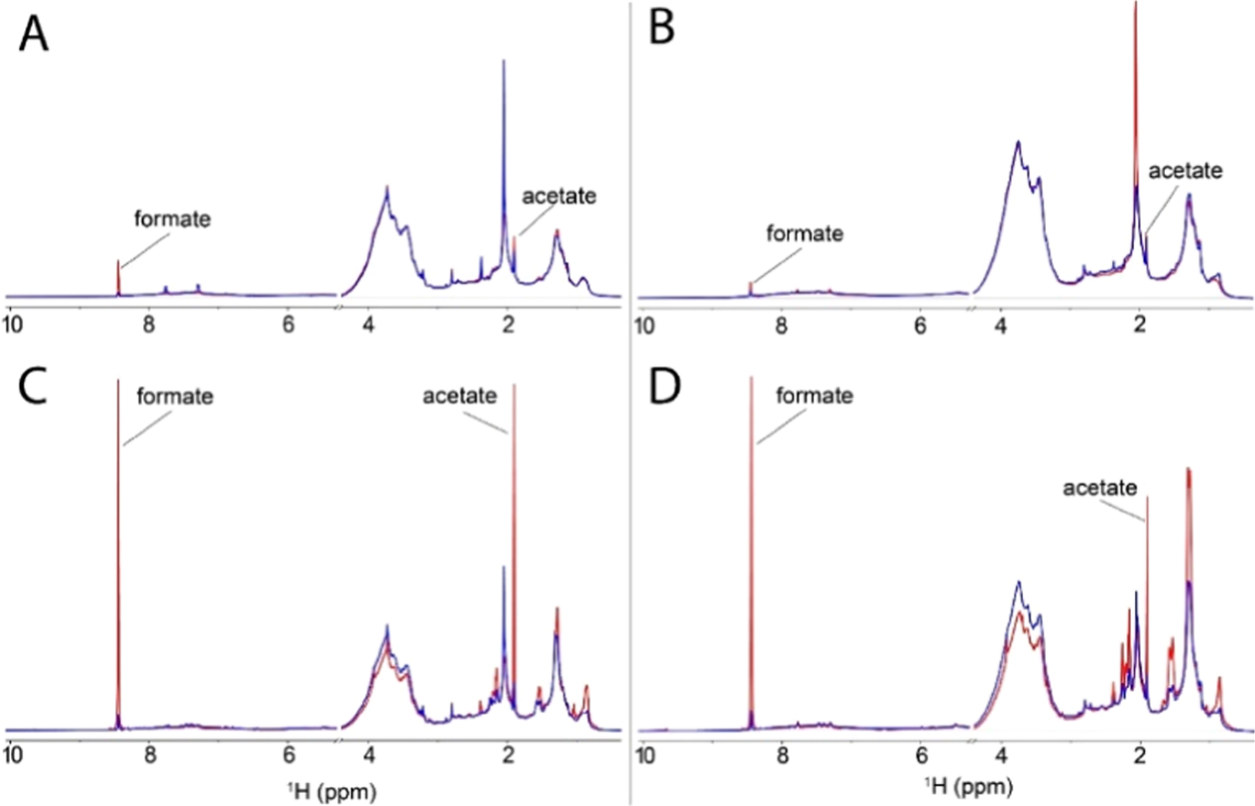
^1^H NMR spectral overlays under dark (blue trace) and light-exposed (red trace) conditions without HNPs for DOM_MS_ (A) and DOM_WS_ (B). Overlays with HNPs for DOM_MS_ (C) and DOM_WS_ (D).

**Figure 4:**
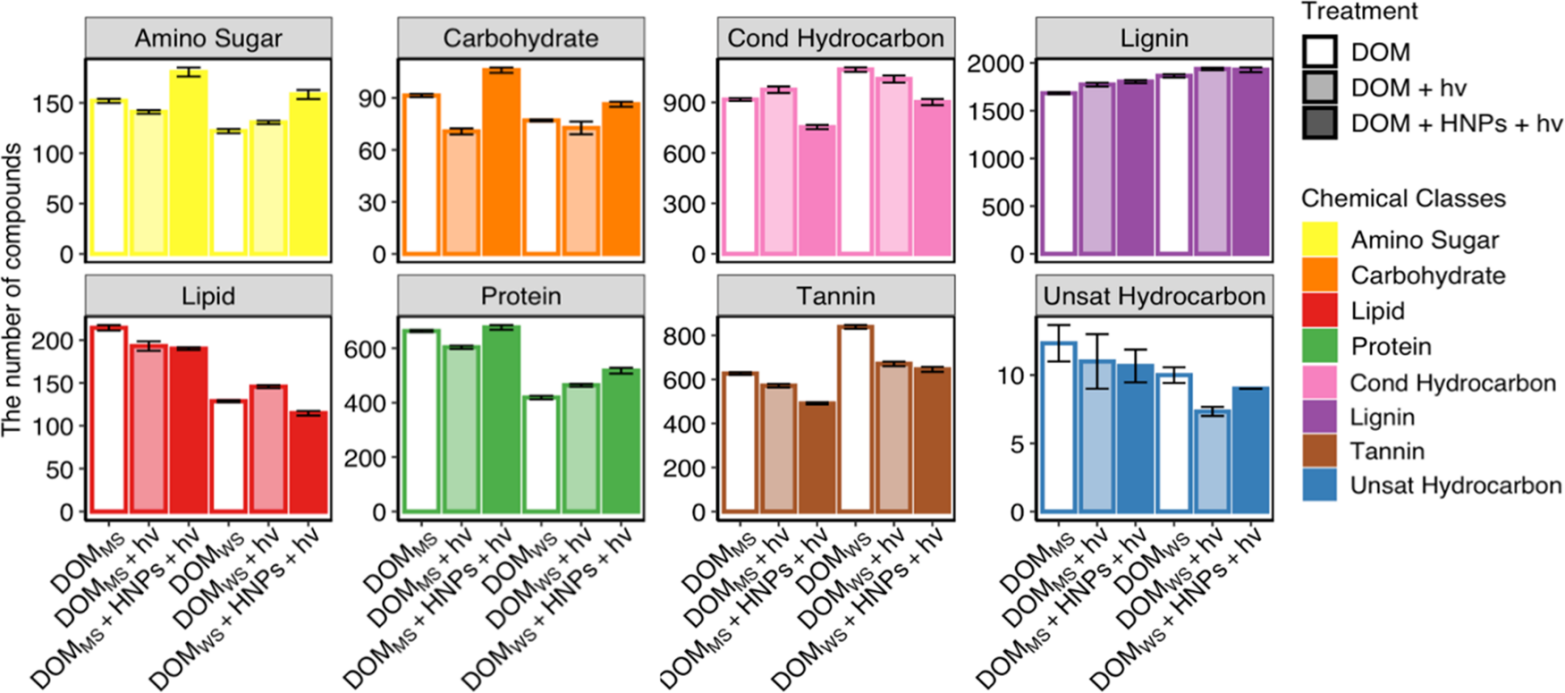
Number of molecules of each chemical class from both aromatic-enriched compounds and aliphatic enriched compounds. DOM is the pristine dissolved organic matter sample extracted from either Michigan soil (DOM_MS_) or Wisconsin soil (DOM_WS_); DOM + *h*ν is the DOM sample with the presence of light; and DOM + HNPs + *h*ν is the DOM sample with the presence of both light and hematite nanoparticles. Error bars represent the standard deviations from three replicates.

Photodegradation of DOM that was catalyzed by the HNPs enhanced the degradation of aromatic compounds but preserved aliphatic compounds, as evidenced by bulk analyses including ^1^H NMR (Figure 3) and FTICR-MS (Figure 4). 1D ^1^H NMR spectroscopy shows that the greatest photolysis-induced change appeared as an increase in the integrated signal intensity in the aromatic region. However, in both DOM_MS_ and DOM_WS_, the aromatic response was largely due to the generation of formate, as the region shows a net decrease when the peak area of formate is subtracted out of each spectrum and then compared (Tables S2 and S3). The relative concentration of aromatic carbon decreased, respectively, 11% and 16% for DOM_MS_ and DOM_WS_ with HNPs and light compared to DOM with light only (Tables S2 and S3). This clearly showed that the aromatic carbon was much more photodegraded with HNPs and light compared with DOM with light only. By comparison, large net increases in signal intensity were observed in the aliphatic regions, especially when compared to the light-exposed samples without HNPs, and these are clearly visible (Figure 3C,D). The functional aliphatic region saw net gains in the integrated area as well as apparent transformations with the decrease in some intensity in the region replaced by the emergence of some sharper features within. Acetate, in particular, showed increases in concentration of 4–10 times that of all the other conditions. Regions seeing net decreases in the integrated area in both DOM samples were the CHO region at around 16–24% as well as the olefinic regions between 15 and 30%. FTICR-MS revealed that, for DOM without HNPs, although the number of aromatic compounds was at times enriched by photolysis, including condensed hydrocarbon-, lignin-, tannin-, and unsaturated hydrocarbon-like molecules, these significantly decreased by 8.15 ± 1.06% (DOM_MS_) and 4.60 ± 1.16% (DOM_WS_) with HNPs versus without (Figure 4C,D and Tables S4 and S5).

The collective results suggest that the addition of HNPs during photolysis preferentially oxidized compounds enriched in aromatic carbon, which was also supported by NMR results. Meanwhile, we found that the number of aliphatic compounds in the DOM, such as amino sugar-, carbohydrate-, lipid-, and protein-like molecules, significantly increased by 14.38 ± 1.30% (DOM_MS_) and 7.86 ± 1.66% (DOM_WS_) with HNPs versus without (Figure 4A,B and Tables S4 and S5).

The presence of both HNPs and light promoted the depolymerization of DOM aromatic macromolecules, most likely due to the production of ^•^O_2_^–^, causing aromatic ring cleavage.^7^ Oxidation by the produced ROS generated new products with a simple aliphatic structure, such as carbohydrates, amino acids, etc., leading to the observed increases in aliphatic compounds. Moreover, HNPs appeared to preserve aliphatic compounds against photodegradation, presumably by mineral–organic matter association. Hydrophilic carbon functional groups, such as carboxylic groups from proteins, may preferentially adsorb to hematite nanoparticle surfaces,^7^ and through stabilization as surface complexes be protected against photodegradation.

Although an ex situ approach, XPS is useful for additional insight into how the speciation of DOM is being modified due to the sensitivity of the C 1s line to carbon speciation. Compositionally, the distributions of carbon species detected by XPS for DOM_WS_ and DOM_MS_ were similar (Table S6). This finding appears not to change significantly when the two DOM samples are adsorbed onto HNPs (Figure S5 and Table S6). Meanwhile, the C 1s XPS spectra of DOM adsorbed to the HNP surface verified that the loss of DOM was not due to adsorption. However, XPS analysis revealed that the presence of both HNP and light leads to a significant increase in the O–C=O concentration, along with a simultaneous decrease in the olefinic/aromatic components. The fraction values of aromatic carbon for DOW_WS_ with light and DOM_WS_ with both HNPs and light were 7.3% and 1.5%, respectively (Table S6). Similarly, the fraction values of aromatic carbon for DOW_MS_ with light and DOM_MS_ with both HNPs and light were 1.1% and 0.0%, respectively (Table S6). This reinforces the notion that the fraction of aromatic carbon was reduced more when HNPs existed under light exposure compared to DOM with light only. The aromatic/olefinic (C=C) and carbonyl (C=O) contents appeared to be slightly higher for DOM_WS_ with HNPs and light; this may indicate a higher fraction of lignin, tannin, or other unsaturated hydrocarbon content in DOM_WS_. When DOM_MS_ was exposed to light in the presence of HNPs, a significant reduction in the unsaturated carbon content and an increase in the =C=O component were observed, which correlates well with bulk analyses by NMR and FTICR-MS.

The presence of light without HNPs also altered DOM composition, but not to the same extent as when HNPs are present. Only small differences are noted between extracts without HNPs upon light exposure from the NMR results. Figure 3A,B shows a decrease in the aromatic region intensity for DOM_WS_ light versus dark with the change in integrated areas not exceeding 8.1%. The fraction of average unsigned changes are 5.0 and 2.5% over all five regions for DOM_WS_ and DOM_MS_, respectively. In agreement with NMR, FTICR-MS also found that the presence of light without HNPs enhanced the degradation of aromatic-enriched compounds in DOM_WS_ but inhibited the degradation of aliphatic-like compounds, compared with unreacted DOM. The number of aromatic-enriched compounds decreased by 4.05 ± 0.93% in the DOM_WS_ in the presence of light but that of aliphatic compounds significantly increased by 8.94 ± 1.28%, compared to that in pristine DOM (Figure 4A,C).

## Environmental Implications

**Scheme 1:**
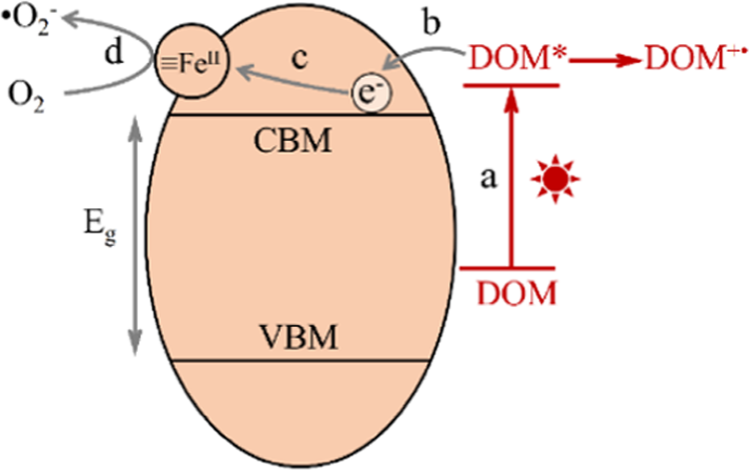
Conceptual Schematic Representation of the Photocatalyzed Mechanism of Bound DOM by Iron Valence Cycling and ROS Generation on the Hematite Surface (a) Chromophoric DOM photosensitization, (b) electron injection from excited chromophoric DOM to the conduction band minimum (CBM) of hematite, (c) creating of ET reactive surface Fe(II)-like cationic sites, and (d) molecular oxygen activation by surface Fe(II) to enhance the production of superoxide radicals. VBM is valence band maximum. “≡” symbol represents the hematite surface.

In the present case, the overall mechanistic model is illustrated in Scheme 1, including key steps of (a) chromophoric DOM photosensitization, (b) electron injection from excited chromophoric DOM to the CBM of hematite, (c) creation of redox-reactive surface Fe(II)-like cationic sites, and (d) molecular oxygen activation by surface Fe(II) to enhance the production of ^•^O_2_^–^. DFT calculations predict that interfacial electron transfer yielding Fe(II) polarons on the hematite (001) surface can reduce dioxygen molecules to superoxide radicals through a one-electron transfer, which is consistent with the ROS being produced, as determined by EPR spectroscopy. ^1^H NMR and ESI-FTICR-MS spectroscopies show that the degradation occurs by depolymerization of the aromatic groups, yielding more aliphatic components, consistent with a progressive loss of the chromophoric content in the DOM (i.e., photobleaching). Hence, one major implication is the importance of low molecular weight organics such as oxalate that can bind to iron and consequently alter the speciation of photogenerated ROS. These results also imply that ROS may potentially provide a useful signature for photosensitizing species that yield natural organic matter degradation at mineral–organic interfaces.

Natural organic matter degradation is foundational to carbon cycling and climate change. In the photic zone of aquatic systems and at the surface of soils, an association of organic carbon with ferric oxide minerals enables poorly understood photocatalytic pathways that can accelerate or redirect its geochemical processing. Using uniformly defined hematite nanoparticles with dissolved organic matter, we show that the photocatalytic ferrous iron pool created at particle surfaces can reduce oxygen to singlet oxygen and superoxide, yielding a robust degradation pathway. These reactive oxygen species could be useful indicators of natural organic matter degradation rates at the Earth’s surface. Generally, this study provides the most comprehensive understanding of the photochemical reactions of DOM with hematite expected in the photic zone of agricultural soils. Further research is needed to elucidate the key relationship between interfacial iron redox cycling and DOM transformation dynamics, with important implications for both geochemical and environmental remediation sciences.

## Supporting Information Available

Synthesis of hematite nanoplatelets; natural DOM extraction; photodegradation experiments; EPR spectroscopy; XPS methods; NMR spectroscopy; FTICR-MS data acquisition and data analysis; free radical analysis; the EPR spectra of standard ROS; the comparison of the photolysis of dissolved organic matter in the presence and absence of hematite nanoplates; the optimized simulation spectra compared to the experimental spectra; intensity changes of corresponding ROS in DEPMPO-trapped EPR; C 1s XPS spectra; EPR parameters; NMR data summary; FTICR-MS data summary; the relative fraction of carbon type of DOM (PDF)
