# Supplementary material for: Photolysis of Dissolved Organic Matter over Hematite Nanoplatelets
Source: Environ Sci Technol. 2024 Jan 31;58(6):2798–807. doi: 10.1021/acs.est.3c08752 (PMC10867828; doi:10.1021/acs.est.3c08752)
Supplement: Supplementary file 1 — es3c08752_si_001.pdf [file es3c08752_si_001.pdf]

Supporting Information (SI) for

Photolysis of Dissolved Organic Matter over Hematite Nanoplatelets

Xiaopeng Huang,[*, a, d, e] *Duo Song*,[a] *Qian Zhao*,[b] *Robert P. Young*,[c] *Ying Chen*,[c] *Eric D. Walter*,[c] *Nabajit Lahiri*,[a] *Sandra D. Taylor*,[a] *Zheming Wang*,[a] *Kirsten S. Hofmockel*,[b] *Fernando Rosario-Ortiz*,[f, g] *Gregory V. Lowry*,[d, e] and *Kevin M. Rosso*[*, a]

[a]Physical and Computational Sciences Directorate, Pacific Northwest National Laboratory, Richland, Washington 99352, United States

[b]Earth and Biological Sciences Directorate, Pacific Northwest National Laboratory, Richland, Washington 99352, United States

[c]Environmental Molecular Sciences Laboratory, Pacific Northwest National Laboratory, Richland, Washington 99352, United States

[d]Civil and Environmental Engineering, Carnegie Mellon University, Pittsburgh, Pennsylvania, 15213, United States

[e]Center for Environmental Implications of Nano Technology (CEINT), Carnegie Mellon University, Pittsburgh, Pennsylvania 15213, United States

[f]Department of Civil, Environmental, and Architectural Engineering, University of Colorado, Boulder, Colorado 80309-0607, United States

[g]Environmental Engineering Program, University of Colorado, Boulder, Colorado 80309-0428, United States

[*]To whom correspondence should be addressed.

(K.M.R) kevin.rosso@pnnl.gov, Phone: +1-509-371-6357; (X.H.) xiaopenh@andrew.cmu.edu

8 texts, 5 figures, and 6 tables.

Text S1. Synthesis of Hematite Nanoplatelets. Briefly, 4.0 mmol of iron chloride was dissolved in 40 mL of absolute ethanol during magnetic stirring. Then, 2.8 mL of water and 3.2 g of sodium acetate were added. After complete dissolution, the mixtures were transferred into a Teflon liner, sealed in a steel vessel and then incubated in an oven at 180 ^{o}C for 12 hours. The harvested sample was thoroughly washed with Nanopure™ water and ethanol, and finally dried at 50 ^{o}C overnight.

Text S2. Natural DOM Extraction. The Wisconsin field site is located at the Arlington Agricultural Research Station of the University of Wisconsin-Madison and the Michigan field site is located at the W.K. Kellogg Biological Station Long-Term Ecological Research Site, USA. Soil was collected from the top 15 cm below the surface, shipped to the laboratory on ice packs, passed through a 2-mm sieve, and air dried at room temperature. Soils from five replicate blocks at each site were composited. Wisconsin soil was characterized as silty loam (containing 66.1±3.6% silt, n=50) while Michigan soil was classified as sandy loam (62.6±12.6% sand, n=50).[1] 4 g of dry soil was weighed in polypropylene centrifuge tubes and mixed with Nanopure^{TM} water at 1:10 w/v ratio. Soil slurry samples were shaken on a horizontal shaker at 100 rpm and 22±1 °C for 16 h and centrifuged at 4000 rpm for 10 min. The supernatant was collected and filtered with 0.2 µm syringe filters, finally yielding dissolved organic matter extracted from Wisconsin soil (DOM_{WS}) and Michigan soil (DOM_{MS}), respectively.

Text S3. Photodegradation Experiments. Briefly, 10 mg of HNPs was dispersed in a 10 mL DOM aqueous solution in a 200 mL cylindrical Pyrex vessel. The mixture was magnetically stirred with a Teflon-coated magnetic stir bar by a magnetic stirrer mixer. The pristine pH of DOM_{WS} and DOM_{MS} were 6.38 and 6.28, respectively. The pH was not altered by the addition of hematite. Therefore, the pH of the mixture was not intentionally adjusted. The suspension was illuminated by a 200 W Xenon arc lamp (UXL-360, HI2608), simulating the sunlight that induces photodegradation reactions. The distance between the reaction vessel and light source was adjusted to be 4 cm. During the

photodegradation process, the temperature of the solution was maintained at room temperature using jacketed glassware and a circulating water-cooling system. The samples were taken out at predetermined time intervals from the vessel with a syringe and filtered with a 0.22 μm PVDF membrane filter.

Text S4. EPR Spectroscopy. A capillary with ID 0.8 mm and OD 1 mm was used to hold the solution in the EPR cavity with both ends sealed by Critoseal. Kinetic measurements were performed by recording EPR spectrum continually before, during and after illumination with a sweep time of 20.97 s and 16 scans (5.6 min per spectrum) at microwave power of 20 mW. The typical settings for the instrument were: microwave frequency = 9.32 GHz, sweep width = 150.0 G, field modulation amplitude = 1.0 G, time constant = 40.96 ms. All EPR simulations were performed using EasySpin 5.2.[2]

To detect transient radicals, these short-loved species were detected using spin trapping. 5-(Diethoxyphosphoryl)-5-methyl-1-pyrroline-N-oxide (DEPMPO)-trapped EPR spectra were obtained because this trap is capable of detecting a variety of oxygen-centered and/or carbon-centered free radicals, such as hydroperoxyl radical (•OOH), hydroxyl radicals (•OH), alkyl radical (•R), alkoxyl radical (•OR), and peroxyl radical (•OOR). Standard control spectra for each of these transient species were collected as reference spectra.

Text S5. XPS Methods. To prepare samples for XPS analyses, DOM-coated HNPs were suspended in deionized water and drop casted on to Si wafers containing a 300 nm layer of thermally grown SiO_2. They were then introduced into the load lock chamber and pumped down to ~1×10^{-8} Torr, then transferred into the analysis chamber operating at a base pressure of 2E-9 Torr. Data acquisition was carried out on an area of 700 μm × 300 μm at normal take-off angles. High-resolution scans were collected at a pass energy, PE, of 40 eV (Full Width at Half Maximum, FWHM, of Au 4f7/2 of metallic

gold at 40 eV PE was 0.8 eV), while survey scans were collected at 160 eV. For the solution-based samples, the supernatant solution was drop casted onto Au-coated Si wafers. The wafers were then transferred into the analysis chamber and analyzed in a similar fashion as described above. All data processing was carried out using CasaXPS software. Gaussian-Lorenztian product forms of lineshapes were used for peak deconvolution, and the FWHM of the C 1s components were constrained to a narrow range of ±0.2 eV. For the solution-based drop-casted DOM extracts, a 'U 3 Tougaard' type background approximation was chosen to model the rising nature of the baseline, while for the DOM-coated HNP samples an iterated Shirley type background approximation was used.

For each of the two DOM samples, high resolution C 1s XPS scans were collected for DOM before and after photolysis, and for DOM adsorbed on HNPs before and after photolysis. For the two DOM samples prior to adsorption on HNPs and prior to photolysis, peak deconvolution of the C 1s region shows that at least six different types of carbon chemical environments, or functional groups, are present. The main reference peak at 285 eV can be attributed to a C-C/C-H chemical state. The feature at ~0.5 eV lower binding energy indicate alkene (C=C) type species. The features at ~1.4 eV and ~2.8 eV higher binding energy were assigned to C-O/C-N and C=O type bonds, respectively, possibly originating from alcohol/amine and carbonyl type species. The feature at ~3.7 eV above the main peak is consistent with ester/carboxylic acid groups (O-C=O), such as from fatty acid or protein-like structures, and the feature at ~4.5 eV above the main peak is consistent with carbonate (CO_3) species on the surface.

Text S6. Nuclear Magnetic Resonance Spectroscopy. Centrifugation and filtration (0.22 μm polyvinylidene fluoride (PVDF) membrane, 25 mm syringe filter) were used to pellet/separate the HNPs from the samples. A volume of 7.0 mL of each sample's supernatant was aliquoted, lyophilized, and reconstituted in 0.550 mL of D_2O (12.7 × concentrated) with 2 % (v/v) of Chenomx internal

standard (IS) solution (5.02 mM sodium trimethylsilylpropanesulfonate (DSS-d6) and 0.2% NaN_3 in 100% D_2O) prior to transferring to 5mm Wilmad glass NMR tubes for measurement. Measurements were conducted at a regulated temperature of 25 °C using a Bruker Avance III spectrometer operating at a field strength of 17.6 T (^1H ν_0 of 750.24 MHz) and equipped with a 5mm Bruker TCI/CP HCN (inverse) cryoprobe with Z-gradient. The W5 WATERGATE ('zggpw5') water suppression pulse sequence [3] was used to acquire all 1D ^1H spectra. Experimental parameters employed included a spectral window of 19.0 ppm, a calibrated 90° pulse width, an acquisition time of 1 s (32k total points), a 45 s relaxation delay, and a total of 128 transients were coadded for each spectrum. The binomial water suppression delay was set to 91 μs thus placing the first nulls outside the signal region at 11.8 and -2.4 ppm. Post-acquisition processing included zero-filling to 64k points, exponential multiplication (1 -5 Hz line-broadening), and semi-automatic, multipoint smooth segments baseline correction using MestReNova version 14.0.1. ^1H spectra were referenced to 0.0 ppm from the methyl signal in the internal DSS-d6 and limited spectral assignments were made using NMRSuite 8.5 Professional (Chenomx) as well as reference to the HMDB and BMRB spectral databases.[4, 5] MestReNova 14.0.1 was used for deconvolution of select peaks for quantitation (acetate and formate) and integration of regions corresponding to key substructures/functional group types based on the chemical shift limits used in Hertkorn et al.[6]

1D ^1H NMR spectroscopy was used to monitor changes in DOM composition by integrating and comparing five specific signal regions representing key organic substructural features using slightly modified ^1H chemical shift , δ_H (ppm), ranges based on Hertkorn et al [6]. The first of the five regions corresponded to pure aliphatic protons (CCCH) from 0.50 – 1.85 ppm. The next region, between 1.85 – 3.10 ppm, encompasses functionalized aliphatics (XCCH, X = O, N, S, or NCH of primary and secondary amines). The region from 3.1 – 4.5 ppm includes oxygenated (OCH) resonances as in

carbohydrates, phenolic methoxy protons, as well as HC_α protons of most amino acids. The region between 4.5 and 5.3 ppm is not included in the integrations due to the suppressed residual proton signal in the deuterated solvent (HDO). The region from 5.3 – 7.0 ppm contains anomeric proton resonances from carbohydrates (O_2CH) at the lower end of the range and olefinic/unsaturated (HC=C) throughout as well as some aromatic resonances, however, the bulk of the aromatic (including heteroaromatics) region is assigned from 7.0 – 10.0 ppm.

Text S7. FTICR-MS Data Acquisition and Data Analysis. DOM samples were diluted in MeOH at a 1:2 ratio to improve ESI efficiency, and then introduced directly to the ESI source with a fused silica tube (30 μm i.d.) at a flow rate of 3.0 μL/min by an Agilent 1200 series pump (Agilent Technologies, Santa Clara CA). The ion accumulation time (IAT) was adjusted between samples to account for different carbon concentrations. In addition, the needle voltage at +4.4 kV; Q1 was set to 50 m/z; and the heated resistively coated glass capillary was operated at 180 °C. DataAnalysis software (BrukerDaltonics version 4.2) converted raw spectra to lists of m/z values, with S/N threshold set to 7, mass measurement error <1 ppm, and absolute intensity threshold to the default value of 100. Chemical formulas were assigned with S/N >7 and the presence of C, H, O, N, S and P and excluding other elements. Van Krevelen diagrams were constructed to assign compounds to the major biochemical classes (i.e., amino sugar-, lipid-, protein-, lignin-, carbohydrate-, tannin-, unsaturated hydrocarbon-, and condensed aromatic-like) based on the assigned chemical formulas. Boundaries of classes on the van Krevelen diagram were based on the ratios of H to C and O to C.[7]

Text S8. DEPMPO-trapped EPR spectra of DOM_{WS} photolysis as a function of illumination time (Figure 1B) showed that the signal increased until reaching a plateau at 11 minutes. A similar trend was also found for DOM_{MS} photolysis (Figure 1C), indicating that the generation of free radicals reached steady state within 11 min. When DOM_{WS} was illuminated, the intensity of spin adducts of

DEPMPO-OOR and DEPMPO-OOH increased within the first 11 minutes and plateaued, while DEPMPO-R plateaued at 1/3 of the intensity of DEPMPO-OOR and DEPMPO-OOH (Figure S4A in the SI). When the SOD was added to DOM_{WS} under illumination, the equilibrium intensity of DEPMPO-OOR and DEPMPO-R both reduced by 2/3, reinforcing that •OOH is the dominant species (Figure S4A in the SI). For DOM_{MS}, the intensity of all spin adducts also plateaued after 11 minutes, with the intensity of DEPMPO-OOR significantly dropping after the addition of SOD (Figure S4B in the SI), further confirming •OOH as the dominant species.

With the addition of HNPs, the in situ EPR also showed that the intensity increased in the first 11 minutes and then reached steady-state for DOM_{WS} (Figure 1D) and DOM_{MS} (Figure 1E). For DOM_{WS} in the presence of HNPs, spin adduct DEPMPO-OR dominated at equilibrium and DEPMPO-R was negligible (Figure S4C in the SI). When SOD was added to the system, DEPMPO-OOR was observed with a much low intensity. The •OR species was not observed with the addition of SOD, indicating that •OOH was the primary free radical while •OR was a secondary free radical (•OOH + R → •OR). As for DOM_{MS} with HNPs (Figure 2E), the intensity increased in the first 17 minutes before plateauing, with DEPMPO-OOR and DEPMPO-OOH dominant at equilibrium and a small amount of DEPMPO-R (Figure S4D in the SI). Therefore, we conclude that hematite nanoplates promoted the generation of •OOH during the photolysis of DOM.

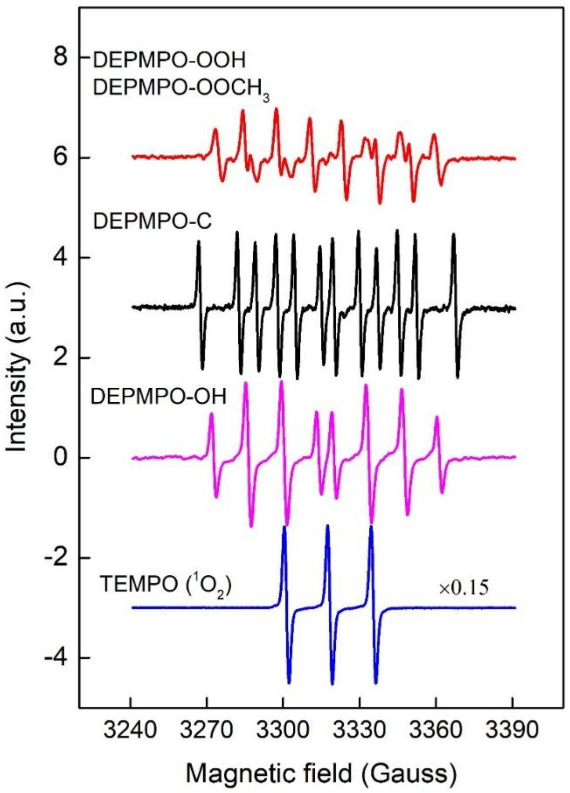

Figure S1. The EPR spectra of standard reactive oxygen species. DEPMPO-OOH and DEPMPO-$OOCH_3$ EPR spectrum was obtained upon UV irradiation of an incubation mixture containing DEPMPO (20 mM), H_2O_2 (3%) and dimethylsulfoxide (DMSO, 10%) in oxygen saturated solution. DEPMPO-C EPR spectrum was obtained upon UV irradiation of an incubation mixture containing DEPMPO (20 mM), H_2O_2 (3%) and DMSO (10%) in deoxygenated solution. DEPMPO-OH EPR spectrum was obtained by photolysis of H_2O_2 (0.5%) in DEPMPO (20 mM) solution. The standard EPR spectrum of singlet oxygen species were obtained from measuring the stable nitroxide radicals 2,2,6,6-tetramethyl-1-piperidinyloxyl (TEMPO, 100 mM) solution.

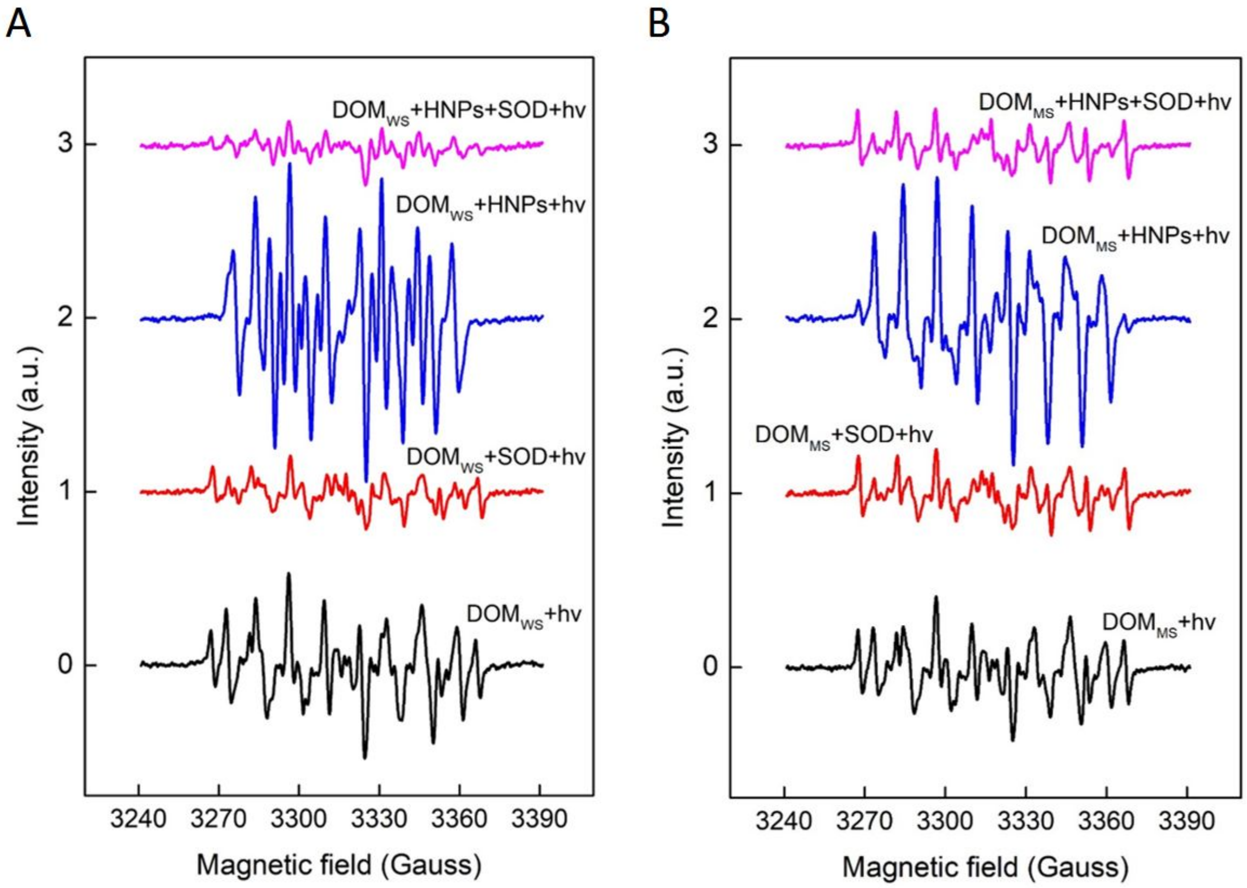

Figure S2. The comparison of the photolysis of dissolved organic matter in the presence and absence of hematite nanoplates. SOD was added to the mixture solutions to remove the •OOH free radicals. Total EPR spectrum was sum of the first four spectrum in Xe lamp illumination with 22 min. All the EPR spectrum was collected in the same parameters. The dosage of SOD and hematite are 0.1 g/L and 1.0 g/L, respectively. The concentration of spin trap DEPMPO is 20 mM. The amount of DOM_{WS} and DOM_{MS} are both 10 μL of the pristine solutions and the pH are 6.38 and 6.28, respectively. Total reaction volume for EPR is 50 μL.

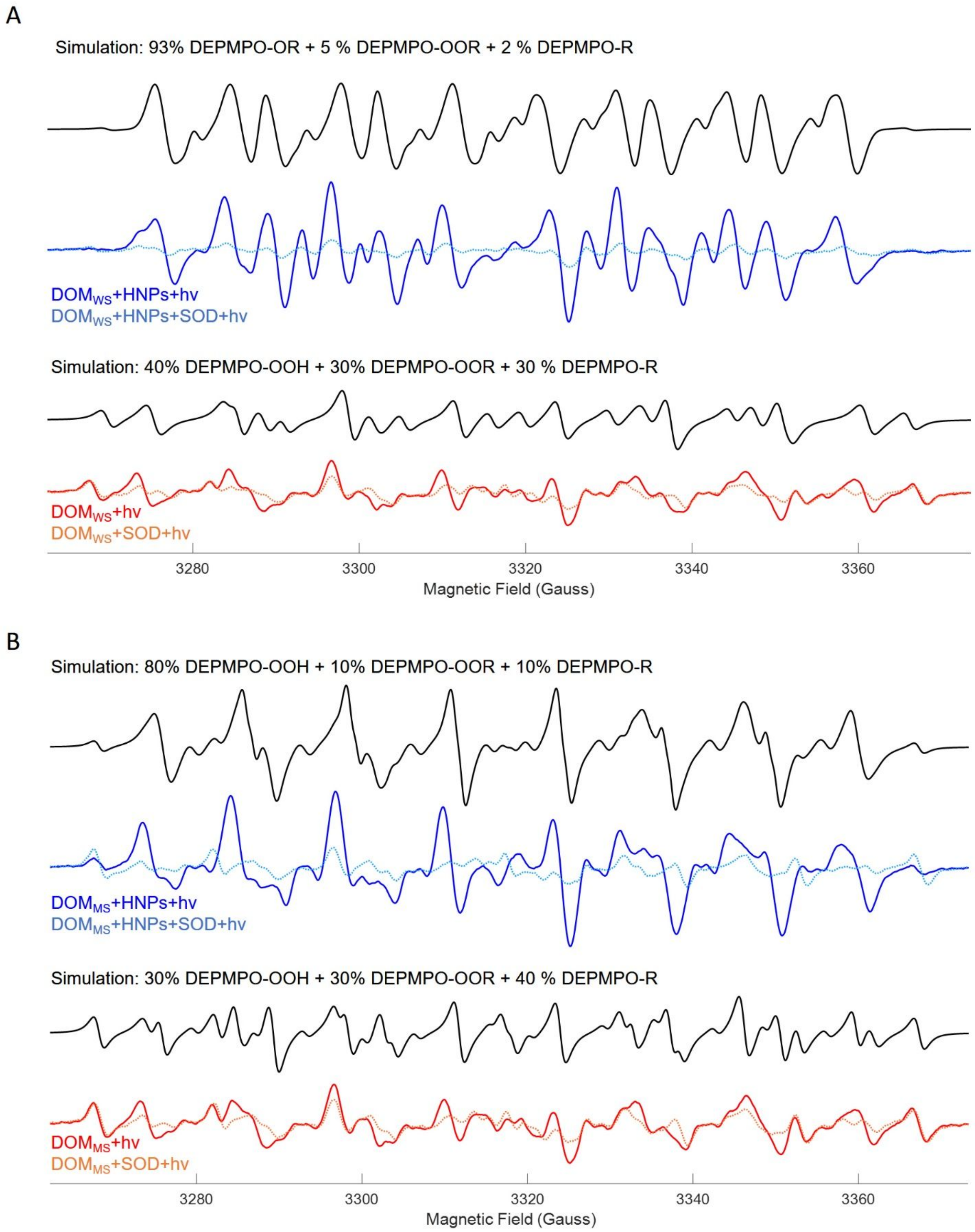

Figure S3. The optimized simulation spectra (black lines) compared to the experimental spectra of the photolysis of dissolved organic matter in the presence (blue/cyan lines) and absence (red/orange lines) of hematite nanoplates.

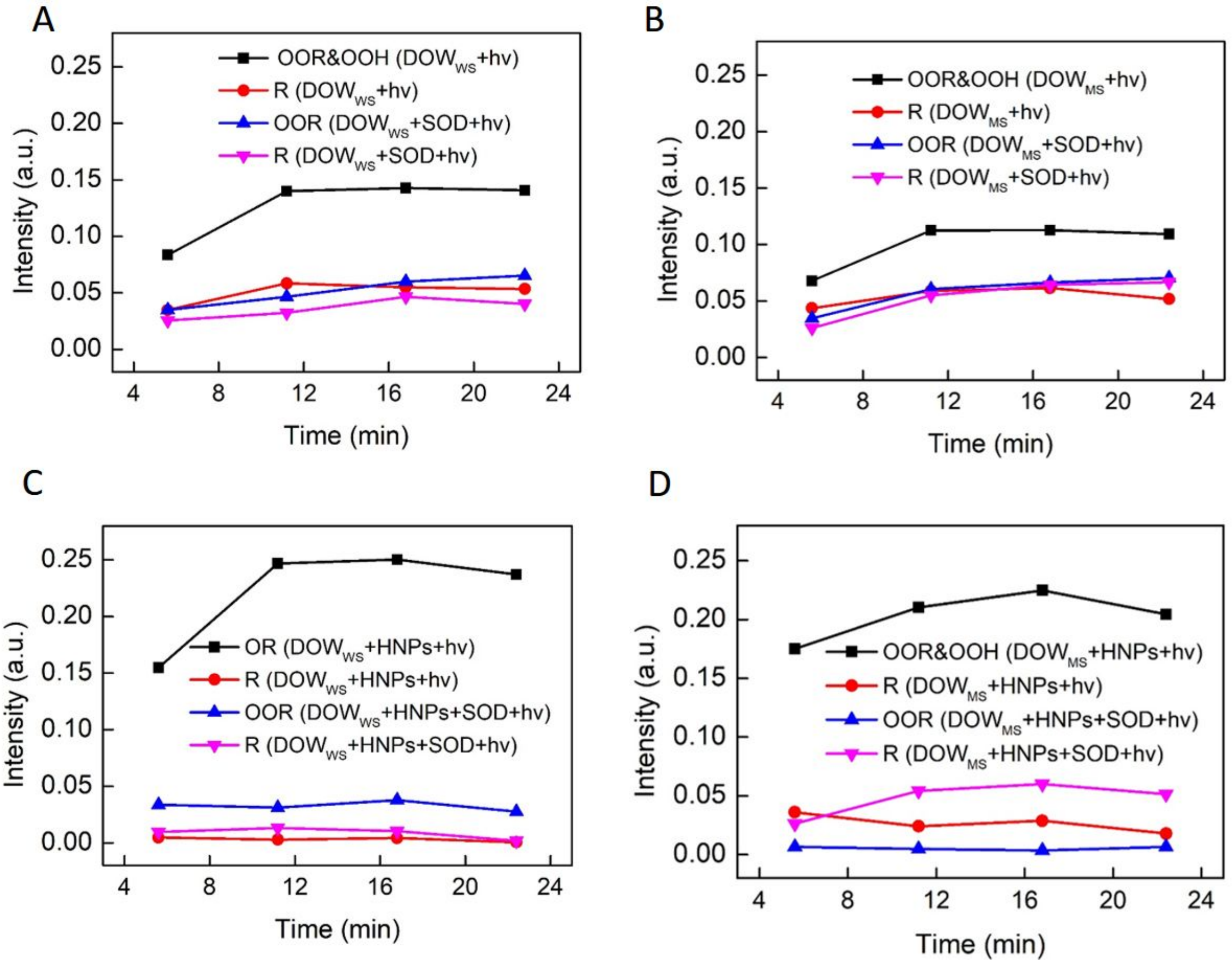

Figure S4. (A) Intensity changes of corresponding ROS in DEPMPO-trapped EPR spectra in the absence and presence of SOD during photolysis of DOM_{WS} as a function of illumination time. (B) Intensity changes of corresponding ROS in DEPMPO-trapped EPR spectra in the absence and presence of SOD during photolysis of DOM_{MS} as a function of illumination time. (C) Intensity changes of corresponding ROS in DEPMPO-trapped EPR spectra in the absence and presence of SOD during photolysis of DOM_{WS} with hematite nanoplates as a function of illumination time. (D) Intensity changes of corresponding ROS in DEPMPO-trapped EPR spectra in the absence and presence of SOD during photolysis of DOM_{MS} with hematite nanoplates as a function of illumination time. The dosage of SOD, and hematite are 0.1 g/L and 1.0 g/L, respectively. The amount of DOM_{WS} and DOM_{MS} are both 10 μL of the pristine solutions and the pH are 6.38 and 6.28, respectively. Total reaction volume for EPR is 50 μL.

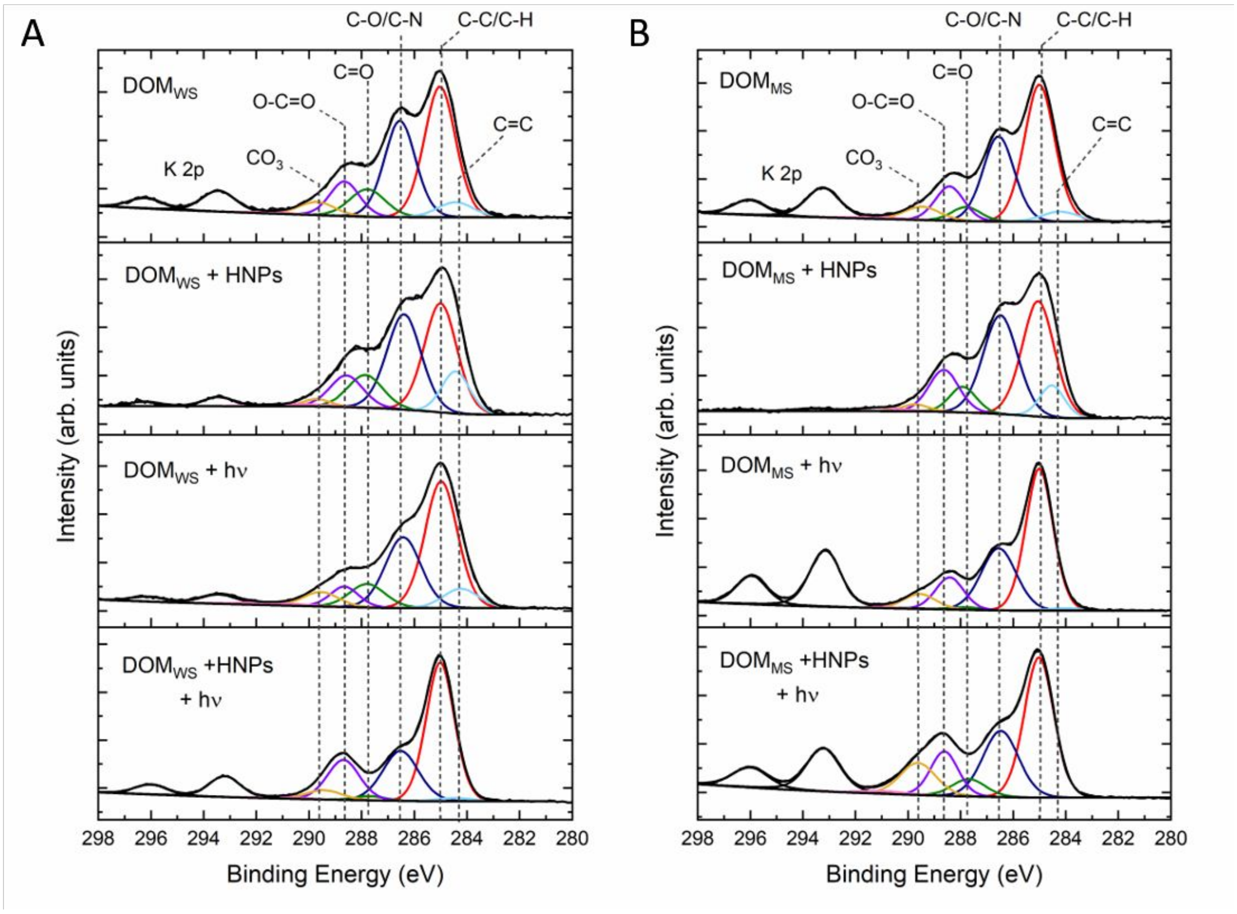

Figure S5. Peak deconvolution of C 1s XPS spectra of dissolved organic matter. (A) C 1s XPS spectra of Wisconsin soil derived DOM (DOM_{WS}). (B) C 1s XPS spectra of Michigan soil derived DOM (DOM_{MS}).

Table S1. EPR parameters of different DEPMPO spin adducts estimated from the best fits to the experimental spectra.

Radical spin adducts	EPR parameters of the spin adduct (g=2.0069)					
	Diastereoisomer	a_H [a] /G [d]	a_N [b]/G	a_P [c]/G	lw [e]	$k_{exchange}$ [f]/s $\times 10^{-7}$
DEPMPO-R		21.3	14.4	49.0	0.13	
DEPMPO-OR	*Trans* 1 (60%)	9.2, 0.9	13.5	46.0	0.19	0
	Trans 2 (40%)	8.2, 1.8	13.7	40.7	0.15	
DEPMPO-OOR	*Trans* 1 (50%)	11.5	13.1	50.0	0.16	1.5
	Trans 2 (50%)	9.0	13.4	48.0		
DEPMPO-OOH	*Trans* 1 (45%)	11.5, 0.9	12.7	50.0	0.12	4.0
	Trans 2 (45%)	9.5, 0.9	12.5	47.0		
	Cis (10%)	9.0	13.4	40.6	0.16	

[a] a_H is the hyperfine coupling constants for proton.

[b] a_N is the hyperfine coupling constants for nitrogen.

[c] a_P is the hyperfine coupling constants for phosphorus.

[d] G is Gauss, the unit of hyperfine constant.

[e] lw is a peak-to-peak (PP) linewidths in mT.

[f] $k_{exchange}$ is the exchange rate constant.

Table S2. DOM_{MS} Major substructure types, chemical shift regions integrated, % total integrated area of region, and the % change in the integrated area for regions calculated as $100(A_{cond1}-A_{cond2})/A_{cond2}$.

		% total integrated Area				% Change in Int. A		
Major substructure types	Chem Shift Range (ppm)	No HNPs		With HNPs		No HNPs Light vs. Dark	With HNPs Light vs. Dark	Light Exposed w/ vs. w/o HNPs
		Dark	Light	Dark	Light			
aromatic	10.00 .. 7.00	3.8	3.7	4.2	6.7	-2.6	59[a] /-27[b]	79[a] / -11[b]
olefinic	7.00 .. 5.30	2.5	2.5	2.9	2.0	-0.39	-30	-20
HCO	4.50 .. 3.10	48.1	49.3	46.5	39.2	2.4	-16	-20
Funct. Aliphatic	3.10 .. 1.85	24.6	23.2	23.0	25.0	-5.5	8.8	7.7
Aliphatic	1.85 .. 0.50	21.0	21.3	23.5	27.0	1.5	15	27

[a]Difference in integrated area with formate peak intensity included or [b]excluded from the aromatic region integral.

Table S3. DOM_{WS} Major substructure types, chemical shift regions integrated, % total integrated area of region, and the % change in the integrated area for regions calculated as $100(A_{cond1}-A_{cond2})/A_{cond2}$.

		% total integrated Area				% Change in Int. A		
Major Substructural Types	Chem. Shift Range (ppm)	No HNPs		With HNPs		No HNPs Light vs. Dark	With HNPs Light vs. Dark	Light with vs. without HNPs
		Dark	Light	Dark	Light			
aromatic	10.00 .. 7.00	4.1	3.7	3.7	5.5	-8.1	48[a] / -16[b]	48[a] / -16[b]
olefinic	7.00 .. 5.30	3.0	2.9	2.9	2.4	-4.9	-17	-15
HCO	4.50 .. 3.10	50.2	50.8	48.8	38.8	1.3	-20	-24
Funct. Aliphatic	3.10 .. 1.85	23.4	22.0	21.9	23.2	-5.4	5.8	5.4
Aliphatic	1.85 .. 0.50	19.4	20.5	22.6	30.0	4.5	34	46

[a]Difference in integrated area with formate peak intensity included or [b]excluded from the aromatic region integral.

Table S4. The number of molecules of each chemical class from both energetically favorable and unfavorable compound groups in Wisconsin DOM. The values are mean of three replicates and values in parenthesis are fractions of the number of molecules in each chemical class to the total number of molecules in a sample.

Wisconsin Soil (DOM_{WS})		No HNPs		With HNPs	No HNPs	Light
Compound Groups	Chemical Classes	Dark	Light	Light	Light - Dark	w/ - w/o HNPs
Energetically favorable compound	Amino Sugar	122.0 (2.68%)	130.7 (2.93%)	158.3 (3.63%)	7.13%	21.12%
	Carbohydrate	77.0 (1.69%)	72.7 (1.63%)	86.3 (1.98%)	-5.58%	18.71%
	Lipid	128.7 (2.83%)	145.7 (3.26%)	114.7 (2.63%)	13.21%	-21.28%
	Protein	418.7 (9.19%)	464.0 (10.39%)	517.7 (11.87%)	10.82%	11.57%
	Sum	746.4 (16.39%)	813.1 (18.20%)	877.0 (20.10%)	8.94%	7.86%
Energetically unfavorable compound	Cond Hydrocarbon	1095.0 (24.04%)	1039.0 (23.26%)	902.3 (20.68%)	-5.11%	-13.16%
	Lignin	1864.7 (40.95%)	1936.7 (43.36%)	1928.3 (44.10%)	3.86%	-0.43%
	Tannin	838.0 (18.40%)	670.3 (15.01%)	645.7 (14.80%)	-20.01%	-3.67%
	Unsat Hydrocarbon	10.0 (0.22%)	7.3 (0.16%)	9.0 (0.21%)	-27.00%	23.29%
	Sum	3807.7 (83.61%)	3653.3 (81.80%)	3485.3 (79.90%)	-4.05%	-4.60%

Table S5. The number of molecules of each chemical class from both energetically favorable and unfavorable compound groups in Michigan DOM. The values are mean of three replicates and values in parenthesis are fractions of the number of molecules in each chemical class to the total number of molecules in a sample.

Michigan Soil (DOM_{MS})		No HNPs		With HNPs	No HNPs	Light
Compound Groups	Chemical Classes	Dark	Light	Light	Light - Dark	w/ - w/o HNPs
Energetically favorable compound	Amino Sugar	152.0 (3.49%)	141.0 (3.25%)	180.7 (4.29%)	-7.24%	28.16%
	Carbohydrate	91.3 (2.09%)	70.7 (1.63%)	106.0 (2.52%)	-22.56%	49.93%
	Lipid	214.3 (4.92%)	193.0 (4.45%)	190.0 (4.51%)	-9.94%	-1.55%
	Protein	663.3 (15.21%)	603.7 (13.91%)	676.7 (16.06%)	-8.99%	12.09%
	Sum	1120.9 (25.71%)	1008.4 (23.23%)	1153.4 (27.37%)	-10.04%	14.38%
Energetically unfavorable compound	Cond Hydrocarbon	917.0 (21.03%)	976.0 (22.48%)	753.7 (17.89%)	6.43%	-22.78%
	Lignin	1683.0 (38.60%)	1774.0 (40.87%)	1805.3 (42.84%)	5.41%	1.76%
	Tannin	626.7 (14.37%)	571.3 (13.16%)	491.0 (11.65%)	-8.84%	-14.06%
	Unsat Hydrocarbon	12.3 (0.28%)	11.0 (0.25%)	10.7 (0.25%)	-10.57%	-2.73%
	Sum	3239.0 (74.29%)	3332.3 (76.77%)	3060.7 (72.63%)	2.88%	-8.15%

Table S6. The relative fraction of carbon type of DOM adsorption and photodegradation onto hematite nanoplatelets.

**Entry**	**Fraction of Carbon Type (100%)**					
	C=C (Aromatic)	C-C, C-H (Aliphatic)	C-O, C-N (Alcohol/Amines)	C=O (Carbonyl)	O=C-O (Ester/Acid)	Carbonates
DOM_{WS}	5.3	40.3	29.9	9.7	10.2	4.7
DOM_{WS}/HNPs, dark	11.3	34.0	31.6	11.0	10.1	1.9
DOM_{WS}+hν	7.3	44.5	27.3	9.1	6.3	5.5
DOM_{WS}+HNPs+hν	1.5	51.6	23.6	1.6	17.2	4.4
DOM_{MS}	3.9	46.2	29.3	4.5	10.9	5.2
DOM_{MS}/HNPs, dark	7.9	37.1	33.7	6.8	12.6	1.8
DOM_{MS}+hν	1.1	51.1	28.4	1.1	11.9	6.3
DOM_{MS}+HNPs+hν	0.0	43.9	24.9	6.1	13.0	12.1

References.

(1) Sanford, G. R.; Oates, L. G.; Jasrotia, P.; Thelen, K. D.; Robertson, G. P.; Jackson, R. D. Comparative productivity of alternative cellulosic bioenergy cropping systems in the North Central USA. *Agric., Ecosyst. Environ.* **2016**, *216*, 344-355.

(2) Stoll, S.; Schweiger, A. EasySpin, a comprehensive software package for spectral simulation and analysis in EPR. *J. Magn. Reson.* **2006**, *178*, 42-55.

(3) Liu, M. L.; Mao, X. A.; Ye, C. H.; Huang, H.; Nicholson, J. K.; Lindon, J. C. Improved WATERGATE pulse sequences for solvent suppression in NMR spectroscopy. *J. Magn. Reson.* **1998**, *132*, 125-129.

(4) Ulrich, E. L.; Akutsu, H.; Doreleijers, J. F.; Harano, Y.; Ioannidis, Y. E.; Lin, J.; Livny, M.; Mading, S.; Maziuk, D.; Miller, Z.; Nakatani, E.; Schulte, C. F.; Tolmie, D. E.; Kent Wenger, R.; Yao, H.; Markley, J. L. BioMagResBank. *Nucleic Acids Res.* **2007**, *36*, D402-D408.

(5) Wishart, D. S.; Tzur, D.; Knox, C.; Eisner, R.; Guo, A. C.; Young, N.; Cheng, D.; Jewell, K.; Arndt, D.; Sawhney, S.; Fung, C.; Nikolai, L.; Lewis, M.; Coutouly, M.-A.; Forsythe, I.; Tang, P.; Shrivastava, S.; Jeroncic, K.; Stothard, P.; Amegbey, G.; Block, D.; Hau, D. D.; Wagner, J.; Miniaci, J.; Clements, M.; Gebremedhin, M.; Guo, N.; Zhang, Y.; Duggan, G. E.; MacInnis, G. D.; Weljie, A. M.; Dowlatabadi, R.; Bamforth, F.; Clive, D.; Greiner, R.; Li, L.; Marrie, T.; Sykes, B. D.; Vogel, H. J.; Querengesser, L. HMDB: the Human Metabolome Database. *Nucleic Acids Res.* **2007**, *35*, D521-D526.

(6) Hertkorn, N.; Harir, M.; Koch, B.; Michalke, B.; Schmitt-Kopplin, P. High-field NMR spectroscopy and FTICR mass spectrometry: powerful discovery tools for the molecular level characterization of marine dissolved organic matter. *Biogeosciences* **2013**, *10*, 1583-1624.

(7) Kim, S.; Kramer, R. W.; Hatcher, P. G. Graphical Method for Analysis of Ultrahigh-Resolution

Broadband Mass Spectra of Natural Organic Matter, the Van Krevelen Diagram. *Anal. Chem.* **2003**, *75*, 5336-5344.
